# Alcohol Consumption during the COVID-19 Pandemic: A Cross-Sectional Survey of US Adults

**DOI:** 10.3390/ijerph17249189

**Published:** 2020-12-09

**Authors:** Elyse R. Grossman, Sara E. Benjamin-Neelon, Susan Sonnenschein

**Affiliations:** 1 Department of Health, Behavior and Society, Johns Hopkins Bloomberg School of Public Health, Baltimore, MD 21205, USA; sara.neelon@jhu.edu; 2 Advancement Strategy Consulting, LLC, Columbia, MD 21046, USA; 3 Psychology Department, University of Maryland, Baltimore County, Baltimore, MD 21250, USA; sonnensc@umbc.edu

**Keywords:** coronavirus, binge drinking, pandemic, adults, public health

## Abstract

Emerging but limited evidence suggests that alcohol consumption has increased during the COVID-19 pandemic. This study assessed: (1) whether drinking behaviors changed during the pandemic; and, (2) how those changes were impacted by COVID-19-related stress. We conducted a cross-sectional online survey with a convenience sample of U.S. adults over 21 years in May 2020. We conducted adjusted linear regressions to assess COVID-19 stress and alcohol consumption, adjusting for gender, race, ethnicity, age, and household income. A total of 832 responded: 84% female, 85% White, and 72% ages 26–49. Participants reported consuming 26.8 alcohol drinks on 12.2 of the past 30 days. One-third of participants (34.1%) reported binge drinking and 7.0% reported extreme binge drinking. Participants who experienced COVID-19-related stress (versus not) reported consuming more drinks (β = 4.7; CI (0.2, 9.1); *p* = 0.040) and a greater number of days drinking (β = 2.4; CI (0.6, 4.1); *p* = 0.007). Additionally, 60% reported increased drinking but 13% reported decreased drinking, compared to pre-COVID-19. Reasons for increased drinking included increased stress (45.7%), increased alcohol availability (34.4%), and boredom (30.1%). Participants who reported being stressed by the pandemic consumed more drinks over a greater number of days, which raises concerns from both an individual and public health perspective.

## 1. Introduction

Pandemics such as COVID-19 can cause many medical, psychological, and sociological problems [1], including increased alcohol consumption and related harms from such consumption [2]. Alcohol is a harmful substance [3], and is, in fact, currently the fourth leading preventable cause of death in the U.S. [4]. Excessive drinking has also been associated with increased violence [5], crime [6], poverty [7], sexually transmitted diseases [8], and other significant public heath harms.

Research shows that those experiencing stress often report increased alcohol consumption and misuse [9]. When individuals experience periods of economic or psychological stress, they often consume more alcohol, resulting in increased symptoms of alcohol abuse and alcohol dependence [2,10]. For example, researchers found that individuals in China who were quarantined or worked in high-risk locations during the 2003 SARS epidemic were more likely to use alcohol as a coping mechanism [2]. This was significantly associated with “increased alcohol abuse/dependence symptoms” three years after the outbreak. Similarly, adults in New York City with posttraumatic stress disorder two years after the 2001 attacks on the World Trade Center also reported increased alcohol use and binge drinking [10]. The emergence of COVID-19 may have similar impacts on alcohol consumption and related harms [11], but the effects are still mainly unknown.

The Centers for Disease Control and Prevention (CDC) were alerted to the first confirmed case of COVID-19 in the U.S. on 22 January 2020; the first reported death occurred on 29 February 2020 in Washington state [12,13]. Due to concern over the contagiousness of COVID-19 and the harm suffered if contracted, the Washington governor declared a state of emergency that same day (29 February 2020) [14]. Most other U.S. states quickly followed suit. By 20 April 2020, all but eight states had issued state-wide shelter-at-home orders requiring residents to stay home unless conducting “essential activities” [15]. Concurrently, many states relaxed their alcohol laws to provide economic support for restaurants and liquor stores. For example, in many states, adults could, for the first time, order beer, wine, spirits—and sometimes even cocktails—for curbside or home delivery [16].

During the seven weeks between 1 March and 18 April 2020, there were large increases in alcohol sales in the U.S. [17]. Data from the week ending 21 March indicated that alcohol sales for off-premise locations (e.g., liquor stores) had increased by 54% and online alcohol sales had increased by 262% compared to sales data from the same week in 2019. Although the increases in alcohol sales did not remain at these levels, overall data for that time period showed that in-store purchases were up by 21% and online alcohol sales by 234% compared to 2019. It is unclear, however, whether individuals had been increasing their alcohol consumption or only stockpiling alcoholic beverages.

Alcohol policy experts have identified two ways that COVID-19 might impact alcohol consumption [11]: (1) alcohol use and related harms may increase due to stress triggered by “financial difficulties, social isolation, and uncertainty about the future”; or, (2) alcohol use and related harms may decrease due to restrictions on the “physical and financial availability (i.e., affordability)” of alcohol. Although one study comparing data on adult alcohol consumption from 28 May to 16 June 2020 to 6 weeks in early 2019 found that the frequency of alcohol consumption increased both overall and for specific demographics [18], it did not examine why people had increased their drinking.

Therefore, to better understand how the COVID-19 pandemic impacted adult alcohol consumption in the U.S., we surveyed a convenience sample in May 2020 to assess rates of current alcohol consumption and the prevalence of binge drinking and extreme binge drinking in the past 30 days during COVID-19 (Aim 1). We also compared current alcohol consumption and the prevalence of binge drinking and extreme binge drinking in the past 30 days between participants who reported being very impacted by COVID-19 versus those who did not (Aim 2). Lastly, we examined whether U.S. adults reported that their alcohol intake had changed in the past 30 days compared to their drinking behaviors prior to COVID-19, and the reasons given for any perceived changes in their consumption (Aim 3).

## 2. Materials and Methods

### 2.1. Design

This study used a cross-sectional design to survey a convenience sample of U.S. adults over the age of 21 in May 2020. The survey asked about alcohol consumption and COVID-19-related stress over the past 30 days, consisted of 61 questions (29 demographics questions, 18 related to alcohol consumption, 14 related to stress or lifestyle changes during COVID-19), and was administered online. The questions on alcohol use were taken from the 2018 National Survey on Drug Use and Health–NSDUH [19] and some of the questions on stress or lifestyle changes during COVID-19 were adapted from The Pandemic Stress Index [20]. This study was reviewed by the University of Maryland, Baltimore County Institutional Review Board who determined that it met the requirements for exemption, as the research only included interactions involving survey procedures where the identity of the human subjects could not be readily ascertained, directly or through identifiers linked to the subjects.

### 2.2. Measures

We used four outcome measures: (1) the number of days in the past 30 days on which alcohol was consumed; (2) the total number of drinks consumed over the past 30 days (calculated by multiplying Outcome 1 by the average quantity of drinks consumed per day); (3) whether participants engaged in binge drinking (i.e., having 4 (for females) or 5 (for males) or more drinks in one sitting at least once over the past 30 days [21]); and (4) whether participants engaged in extreme binge drinking (i.e., binge drinking 10 or more times) over the past 30 days.

The primary exposure variable came from a question that asked: “How much is/did COVID-19 (Coronavirus) impact your day-to-day life?” with the five answer choices collapsed into two groups “0” (“Not at all”; “A little”; or “Much”) and “1” (“Very much”; “Extremely”). Participants were then asked two questions about how COVID-19 had impacted their life and given multiple answer choices plus blank spaces to add any additional answers and told to “Check all that apply”.

Participants were also asked if their drinking behaviors had increased, decreased, or stayed the same when compared to their drinking behaviors pre-COVID-19. Participants who reported increased intake were asked why and given eight answer choices plus a blank space to add any additional answers and told to “Check all that apply”. The eight answer choices fell into three categories: (1) increased stress—from distance learning, family, finances, and being confined to the home; (2) increased alcohol availability—because alcohol was readily available at home or easy to get delivered; and (3) boredom. Similarly, participants who reported decreased intake were also asked why and given six answer choices plus a blank space and told to “Check all that apply”. The six answer choices fell into three categories: (1) decreased alcohol availability—because there was less alcohol available at home or because participants could not go out to restaurants or bars; (2) less free time—because participants needed to care for their children, care for sick family members, or spend more time working; and (3) less available money to spend on alcohol. No follow-up questions were asked of those participants who reported no change in their alcohol consumption.

### 2.3. Participants

We recruited a convenience sample of participants from across the U.S. through social media posts (two on Facebook, three on Twitter, and one on Instagram) and emails sent via group listservs (two emails through the U.S. Alcohol Policy Alliance and one email through the Alcohol, Tobacco, and Other Drugs’ section of the American Public Health Association) in May 2020 and offered participants the chance to win one of fifteen $25 Amazon gift cards. The posts and emails were then shared or distributed through snowball sampling [22]. Thus, we were not able to trace all contacts with potential participants or link completed surveys with a specific recruitment method. Social media posts and emails contained a link to complete the survey. Participants who said they were under the age of 21 years or who did not consent to continue with the survey after reading information about the study were immediately exited from the online survey.

All subjects gave their informed consent for inclusion before they participated in the study. The study was conducted in accordance with the Declaration of Helsinki, and the protocol was approved by the Ethics Committee of the University of Maryland, Baltimore County (Protocol #: Y20SS20205).

### 2.4. Analyses

We conducted unadjusted *t*-tests, χ^2^ tests, and ANOVAs to test for differences in alcohol consumption between demographic groups, including gender, race, ethnicity, household income, and households with children. We then conducted adjusted analyses, selecting covariates a priori based on prior literature [23,24,25]. They included gender, race, ethnicity, age, and household income. We performed two separate adjusted linear regressions to assess whether the primary exposure variable (a binary variable indicating “0” for less stress due to COVID-19 and “1” for more stress due to COVID-19) was associated with the number of days alcohol was consumed or the total number of drinks consumed over the past 30 days. In a sensitivity analysis, we conducted Poisson regressions examining the number of days out of the past 30 days on which alcohol was consumed and the total number of drinks consumed over the past 30 days. We then performed two separate adjusted logistic regressions to determine whether the primary exposure variable predicted the likelihood of the binary outcomes of engaging in binge drinking at least once or engaging in extreme binge drinking over the past 30 days. Consistent with other studies [26], we excluded data from 11 participants whose reported total alcohol drinks consumed was greater than three standard deviations above the mean. We conducted analyses using Stata 16.1 (StataCorp LLC, College Station, TX, USA) with a significance level of 0.05. We present results in terms of parameter estimates, 95% confidence intervals (CI), and two-sided *p* values.

## 3. Results

### 3.1. COVID-19 Survey Demographics

The survey screener question asking if participants were 21 years of age or older was completed by 998 participants. After cleaning the data—which included removing those who did not pass the screener question, did not consent to participate, or whose alcohol consumption was greater than three standard deviations above the mean—the final sample included data from 832 participants. The majority were female (84.4%), White (84.9%), between the ages of 26 and 49 (72.3%), and had a household income greater than $100,000 USD (67.0%) (Table 1). Fewer than half of the sample (45.1%) had children under the age of 18 currently living with them in the home.

### 3.2. Participants’ Rates of Alcohol Consumption and Prevalence of Binge Drinking and Extreme Binge Drinking during COVID-19

Most of the participants (91.7%) had consumed alcohol within the past year, with 80% having consumed it within the past 30 days. Participants reported consuming alcohol on a mean (standard deviation) of 12.2 (10.3) days and consuming a mean of 26.8 (24.7) alcohol drinks over the past 30 days (Table 2). Of those who consumed alcohol over the past 30 days, 34.1% reported binge drinking at least once and 7.0% reported extreme binge drinking over the past 30 days.

There were no differences between male and female participants in mean number of days consumed alcohol (12.1 days vs. 11.8 days, *p* = 0.810), total drinks consumed (29.6 drinks vs. 26.1 drinks, *p* = 0.246), or percentages reporting binge drinking at least once (36.9% vs. 33.1%, *p* = 0.491) or extreme binge drinking (9.5% vs. 6.5%, *p* = 0.320). Similarly, there were no differences between White and other race participants in mean number of days consumed alcohol (12.3 days vs. 10.7 days, *p* = 0.166), total drinks consumed (26.9 drinks vs. 25.7 drinks, *p* = 0.686), or percentages reporting binge drinking at least once (32.4% vs. 41.3%, χ^2^ *p* = 0.119). However, other race participants reported engaging in extreme binge drinking significantly more often than White participants (13.8% vs. 5.9%, χ^2^ *p* = 0.010). Participants with children under the age of 18 currently living with them reported consuming alcohol on a significantly greater mean number of days (13.0 days vs. 11.6 days, *p* = 0.054). There were no other significant differences between participants with children and those without children in total drinks consumed (27.8 drinks vs. 26.0 drinks, *p* = 0.337), percentages reported binge drinking at least once (33.9% vs. 34.2%, *p* = 0.958), or percentages reported extreme binge drinking (7.3% vs. 6.7%, *p* = 0.777).

### 3.3. Comparison of Alcohol Consumption, Binge Drinking, and Extreme Drinking between Participants Who Reported Stress during COVID-19 Versus Those Who Did Not

Participants who reported experiencing “very much” or “extreme” stress due to COVID-19 also reported consuming significantly more alcohol than participants who did not report these high levels of stress. The adjusted linear regression model showed an increase in the mean number of days on which alcohol was consumed compared to non-stressed participants during COVID-19 (β = 2.3 days; CI (0.6, 4.1); *p* = 0.009). In other words, stressed participants consumed alcohol on 2.3 more days over the past 30 days than non-stressed participants. It also showed an increase in the number of alcohol drinks consumed over the past 30 days by stressed participants, with these participants reporting consuming 4.6 more drinks than non-stressed participants (β = 4.6 drinks; CI (0.2, 9.1); *p* = 0.043). In the sensitivity analyses, direction and significance of results did not change (data not shown). There was no significant difference between these two groups in terms of likelihood of reporting engaging in binge drinking at least once or extreme binge drinking over the past 30 days. Top concerns during COVID-19 for respondents, included having to socially distance (76.5%); having less time to spend with family members and friends (60.1%); and schools and daycares being closed (47.1%).

### 3.4. Participants’ Perceptions of Drinking Patterns Pre- and Post-COVID-19

In an unadjusted analysis, almost two-thirds of 2020 participants (60.1%) reported that their drinking had increased compared to before COVID-19 (Table 3). Of those, 45.7% reported that their drinking had increased because of increased stress, 34.4% reported that their drinking had increased because of the increased availability of alcohol, and 30.1% reported that their drinking had increased because of boredom. Almost two-thirds of the participants (63.7%) listed some combination of these three reasons. The remainder either gave no reason (1.7%) or some other reason (4.0%), such as “It gives me the feeling of going out”, “I feel safer because I am at home”, “It’s a tasty distraction”, or “It feels permissible”.

Of the full sample, 12.8% reported that their drinking had decreased and 27.0% reported that there had been no change in their drinking behavior pre- and post-COVID-19. Over half (58.1%) reported that their drinking had decreased because of diminished alcohol availability, 29.7% reported that their drinking had decreased because of less free time, and 12.2% reported that their drinking had decreased because of having less money. About one-fifth (21.6%) listed some combination of these three reasons and the remainder gave some other reason (25.7%). These included attempts to diet or adhere to a healthier lifestyle (e.g., “I want to stay healthy and conserve my body’s energy to fight off COVID, should I become infected”); concerns over the impact of alcohol on mental health (e.g., “Drinking amplifies my emotions, including anxiety” and “Struggling with Depression/Anxiety from COVID-Abstained from alcohol as a result”); and decreased interest in alcohol consumption (e.g., “less interested, not as social, drinking is less enjoyable when locked up in house”).

There were also demographic differences between those participants who reported that their drinking had increased, decreased, and stayed the same. For example, more participants with children under the age of 18 currently living with them reported that their drinking had increased (53.6%) than had decreased (32.4%) or stayed the same (35.9%, χ^2^ *p* < 0.001). In addition, a larger percentage of males reported that their drinking had decreased (29.17%) compared to those whose drinking had increased (13.1%) or stayed the same (12.6%, χ^2^ *p* = 0.002). And finally, 51.4% of those whose drinking had decreased also reported being stressed by having to spend more time working versus 43.6% of those whose drinking had increased and 30.9% of those whose drinking had stayed the same (χ^2^ *p* = 0.005).

## 4. Discussion

Participants during COVID-19 reported consuming alcohol on an average of 12.2 days and 26.8 alcohol drinks over the past 30 days. Over a third (34.1%) reported engaging in binge drinking and seven percent reported engaging in extreme binge drinking. Those participants who reported being very or extremely impacted by COVID-19, consumed more alcohol (including both on more days and more total drinks) in the past 30 days. Moreover, nearly two-thirds of the participants reported that their drinking had increased compared to their consumption rates prior to COVID-19. Reasons for this increase were increased stress, increased alcohol availability, and boredom.

To put the first aim in context, according to data from the 2018 NSDUH [19], U.S. adults in 2018 consumed alcohol on an average of 4.8 days and 12.0 alcohol drinks over the past 30 days. Almost a third (31.8%) reported engaging in binge drinking and 3.7% reported engaging in extreme binge drinking. From a preliminary comparison, it appears that participants are consuming more alcohol during COVID-19 than in 2019, but more research is warranted. If this is correct, it would support the first hypothesis posited by alcohol policy experts [11] that alcohol consumption would increase during COVID-19, due, in part, to stress.

Other studies from across the world have also found some increases in alcohol consumption during COVID-19. For example, Pollard et al. used a subset (*n* = 1540) of a nationally representative, probability-sampled panel of 6000 U.S. adults ages 18 years and older to compare alcohol consumption in 2020 (28 May to 16 June) to 2019 (29 April to 9 June) [18]. They found that the frequency of alcohol consumption increased both overall and for specific demographics. Additionally, an online survey of over 1000 mostly female young adults in Poland found that 14.6% reported an increase in alcohol consumption [27]. The remainder of the sample either did not report any increase in drinking during quarantine (77%) or were uncertain whether their consumption was affected (8.3%). A survey of 182 patients with pre-existing alcohol disorders registered in an alcohol clinic in London, England, found that being in lockdown was “a risk factor for increasing alcohol consumption in people with alcohol use disorders and relapse for those who were previously abstinent” [28]. A study of 13,829 Australians found that one in five adults reported drinking more alcohol during COVID-19 compared to pre-COVID-19 [29]. The current study expands on these previous studies to better understand the impact of COVID-19 on alcohol consumption in the U.S.

The findings from this study help explain the trends observed in U.S. alcohol sales data between March and mid-April [17]. From the results of this study, it appears that U.S. adults have not only purchased more alcohol during COVID-19, but they also consumed more. This is especially concerning because, according to the World Health Organization, “alcohol consumption is associated with a range of communicable and noncommunicable diseases and mental health disorders, which can make a person more vulnerable to COVID-19” [30]. Beyond the increased risk to susceptibility to COVID-19, research has shown that consuming more alcohol is related—and in some cases attributable—to experiencing more alcohol-attributable harms in both the short-term (e.g., injuries from falls or burns) and long-term (e.g., developing liver cirrhosis or cancer) [3]. If we continue to follow the path of others after periods of economic and psychological stress (e.g., the occurrences of natural disasters [31], disease outbreaks [9], and terrorist attacks [10]), then we should expect to see an increase in rates of alcohol misuse and dependence in two to three years.

Unfortunately, the U.S. healthcare system is already overwhelmed due to COVID-19 [32]. Yet a review of emergency department (ED) visits in a large Midwest U.S. healthcare system found that the number of alcohol-related complaints, as a percentage of total behavioral health ED visits, increased from 28.2% to 33.5% [33]. The increase in alcohol consumption observed in this study is concerning as the already strained U.S. healthcare system may not be able to continue responding to people who have alcohol-related emergencies.

Additionally, during the COVID-19 pandemic, states tended to prioritize the economic concerns of restaurants and related businesses and may have inadvertently increased availability and access to alcohol. However, the public health data are conclusive that when states increase availability and access to alcohol, e.g., by adding more stores or extending days and hours of sale, then alcohol consumption and related harm also increase [34,35]. This study demonstrates that over a third of participants reported that their alcohol consumption had increased due to increased availability of alcohol during COVID-19. States should consider such data when making decisions about the strength and severity of their alcohol laws during future public heath emergencies.

However, although almost two-thirds of the sample reported that their alcohol consumption had increased during COVID-19, it should be noted that 12.8% of the participants reported that their alcohol consumption had decreased. One factor in their decreased consumption might be that a larger percentage of these individuals were males without children. Future research should examine the impact of having children currently in the home on parental alcohol consumption as this may help direct public health messaging. These participants who reported decreased alcohol consumption were also more likely to report being stressed by having to spend more time working which could have left less time for alcohol consumption. Lastly, it may be that COVID-19 restrictions or some other issue is related to the decrease in consumption. Future research should examine this.

This study has limitations that may affect the interpretation of the data. The survey was a convenience sample recruited through social media and listservs. As such, we were not able to document all contacts with potential participants. We were also not able to link completed surveys with a specific recruitment method. Both are limitations of snowball sampling [22]. The convenience sample also substantially limits generalizability and, thus, results should be interpreted with this in mind. The sample was mostly female and White. However, the demographics of participants is consistent with what has been found in other online surveys [36]. Even so, these findings may not necessarily be representative of other demographic groups within the U.S. Moreover, the fact that a majority of the sample was under the age of 50 and middle- or upper-income (i.e., making above $100,000) may be associated with possible greater flexibility in their work arrangements [37], which, in turn, may lead to differences in alcohol consumption. Second, although most participants who accessed the survey completed it (82.53%) there may be some differences between those who completed it versus those who did not. Third, the data on alcohol consumption are self-reported and may therefore be an under- or over-estimate. In general, however, research shows that drinkers tend to underestimate their consumption [38]. Fourth, we do not know how many individuals received the survey through social media but opted not to participate. Fifth, participants’ geographic locations were not included in the analyses, and differences in state and local laws regarding COVID-19 lockdowns and shutdowns may be influencing alcohol consumption. Future research should study the impact of differing state laws on adult alcohol consumption. Despite these limitations, these data significantly contribute to our knowledge of alcohol consumption during COVID-19.

## 5. Conclusions

In sum, alcohol use in the U.S. is a public health problem that appears to have worsened since the onset of COVID-19. Adults during COVID-19 reported high levels of alcohol consumption, with those who reported high levels of impact from COVID-19 reporting significantly more alcohol (both more days and total drinks) than participants who were not as impacted by COVID-19. Additionally, participants reported perceived increases in their current alcohol intake compared to pre-COVID-19. Given the findings of this and similar studies, it is important for states to consider both economic and public health concerns when making decisions on U.S. alcohol policy in order to protect individuals, their families, and their communities from the longer-term impacts of increased alcohol intake.

## Figures and Tables

**Table 1:** Demographics from Alcohol Use & COVID-19 Survey, 2020 (*n* = 832).

Demographics	Percent (Number)
Gender	
Female	84.4% (593)
Male	15.7% (110)
Race	
White	84.9% (597)
Other race	15.1% (106)
Hispanic/Latinx Ethnicity	6.7% (47)
Age in Years	
21–25	3.5% (24)
26–34	21.2% (147)
35–49	51.1% (355)
50–64	16.0% (111)
65 or older	8.4% (58)
Household Income, USD	
Less than $39,999	6.8% (47)
$40,000 to $79,999	16.0% (110)
$80,000 to $99,999	10.1% (69)
$100,000 to $149,999	25.1% (172)
$150,000 to $199,999	18.5% (127)
More than $200,000	23.4% (160)
Had Children	
Yes	45.1% (361)
No	54.9% (439)

**Table 2:** Alcohol Consumption and Frequency over Past 30 Days, 2020 (*n* = 832).

**Alcohol**	**Mean (SD)**
Number of Days Consumed Alcohol	12.2 (10.3)
Total Number of Drinks Consumed	26.8 (24.7)
	**Percent (Number)**
Binge Drinking Days-At Least Once	34.1% (205)
Extreme Binge Drinking Over Past 30 Days	7.0% (42)

**Table 3:** Participants’ Perceptions of Drinking Pre- to Post-COVID-19, 2020 (*n* = 832).

Drinking Changes	Percent (Number)
Drinking Increased Compared to Pre-COVID-19	60.1% (347)
Drinking Decreased Compared to Pre-COVID-19	12.8% (74)
Drinking Did Not Change Compared to Pre-COVID-19	27.0% (156)
Reasons for Drinking Increases *	
Increased Stress	45.7% (259)
Increased Alcohol Availability	34.4% (195)
Boredom	30.1% (173)
No Reason Given	1.7% (10)
Other Reason	4.0% (23)
Reasons for Drinking Decreases *	
Diminished Alcohol Availability	58.1% (43)
Less Free Time	29.7% (22)
Less Money	12.2% (9)
Other Reason	25.7% (19)

* Percentages do not add up to 100 percent as participants could choose more than one answer.

## References

[1] Pfefferbaum B., North C.S. (2020). Perspective: Mental health and COVID-19 pandemic. N. Engl. J. Med..

[2] Wu P., Liu X., Fang Y., Fan B., Fuller C.J., Guan Z., Yao Z., Kong J., Lu J., Litvak I.J. (2008). Alcohol abuse/dependence symptoms among hospital employees exposed to a SARS outbreak. Alcohol Alcohol..

[3] GBD 2016 Alcohol Collaborators (2018). Alcohol use and burden for 195 countries and territories, 1990–2016: A systematic analysis for the Global Burden of Disease Study 2016. Lancet.

[4] Stahre M., Roeber J., Kanny D., Brewer R.D., Zhang X. (2014). Contribution of excessive alcohol consumption to deaths and years of potential life lost in the United States. Prev. Chronic Dis..

[5] Snowden A.J. (2019). Exploring violence: The role of neighborhood characteristics, alcohol outlets, and other micro-places. Soc. Sci. Res..

[6] Toomey T.L., Erickson D.J., Carlin B.P., Lenk K.M., Quick H.S., Jones A.M., Harwood E.M. (2012). The association between density of alcohol establishments and violent crime within urban neighborhoods. Alcohol. Clin. Exp. Res..

[7] Khana S., Murray R.P., Barnes G.E. (2002). A structural equation model of the effect of poverty and unemployment on alcohol abuse. Addict. Behav..

[8] Cook R.L., Duncan C.B. (2005). Is there an association between alcohol consumption and sexually transmitted diseases? A systematic review. Sex. Transm. Dis..

[9] Keyes K.M., Hatzenbuehler M.I., Grant B.F., Hasin D.S. (2012). Stress and alcohol: Epidemiologic evidence. Alcohol Res. Curr. Rev..

[10] Boscarino J.A., Kirchner H.L., Hoffman S.N., Sarorius J., Adams R.E. (2011). PTSD and alcohol use after the World Trade Center attacks: A longitudinal study. J. Trauma Stress.

[11] Rehm J., Kilian C., Ferreira-Borges C., Jernigan D., Monteiro M., Parry C.D.H., Sanchez Z.M., Manthey J. (2020). Alcohol use in the times of the COVID-10: Implications for monitoring and policy. Drug Alcohol Rev..

[12] Centers for Disease Control and Prevention (CDC) CDC, Washington State Report First COVID-19 Death. https://www.cdc/media/releases/2020/s0229-COVID-19-first-death.html.

[13] Stokes E.K., Zambrano L.D., Anderson K.N., Marder E.P., Raz K.M., Felix S.E.B., Tie Y., Fullerton K.E. (2020). Coronavirus Disease 2019 case surveillance–United States, January 22–May 30, 2020. MMWR Morb. Mortal. Wkly. Rep..

[14] Inslee J. (2020). Proclamation by the Governor 20-05. https://www.governor.wa.gov/sites/default/files/20-05%20Coronavirus%20%28final%29.pdf?utm_medium=email&utm_source=govdelivery.

[15] Mervosh S., Lu D., Swales V. See Which States and Cities Have Told Residents to Stay at Home. New York Times.

[16] Davis E. States Boost Hospitality Industry with Booze Delivery and Takeout Sales. US News & World Report.

[17] The Nielsen Company (2020). Rebalancing the ‘COVID-19 Effect’ on Alcohol Sales. https://www.nielsen.com/us/en/insights/article/2020/rebalancing-the-covid-19-effect-on-alcohol-sales/.

[18] Pollard M.S., Tucker J.S., Green H.D. (2020). Changes in Adult Alcohol Use and Consequences during the COVID-19 Pandemic in the U.S. JAMA Netw. Open.

[19] Substance Abuse and Mental Health Services Administration (SAMHSA) (2019). 2018 National Survey on Drug Use and Health: Methodological Summary and Definitions.

[20] Harkness A. (2020). The Pandemic Stress Index (PSI).

[21] Center for Behavioral Health Statistics and Quality (CBHSQ) (2016). Key Substance Use and Mental Health Indicators in the United States: Results from the 2015 National Survey on Drug Use and Health (HHS Publication No. SMA 16-4984, NSDUH Series H-51).

[22] Goodman L.A. (1961). Snowball Sampling. Ann. Math. Stat..

[23] Chartier K., Caetano R. (2010). Ethnicity and health disparities in alcohol research. Alcohol Res. Curr. Rev..

[24] National Institute on Alcohol Abuse and Alcoholism (NIAAA) (2019). Alcohol and the Hispanic Community. https://www.niaaa.nih.gov/sites/default/files/hispanicFact.pdf.

[25] Wilsnack R.W., Wilsnack S.C., Kristjanson A.F., Vogeltanz-Holm N.D., Gmel G. (2009). Gender and alcohol consumption: Patterns from the Multinational GENACIS Project. Addiction.

[26] Osborne J.W., Overbay A. (2004). The power of outliers (and why researchers should ALWAYS check for them). Pract. Assess. Res. Eval..

[27] Sidor A., Rzymski P. (2020). Dietary choices and habits during COVID-19 lockdown: Experience from Poland. Nutrients.

[28] Kim J.U., Majid A., Judge R., Crook P., Nathwani R., Selvapatt N., Lovendoski J., Manousou P., Thursz M., Dhar A. (2020). Effect of COVID-19 lockdown on alcohol consumption in patients with pre-existing alcohol use disorder. Lancet Gastroenterol. Hepatol..

[29] Tran T.D., Hammarberg K., Kirkman M., Minh H.T., Nguyen J.F. (2020). Alcohol use and mental health status during the first months of COVID-19 pandemic in Australia. J. Affect. Disord..

[30] World Health Organization (WHO) (2020). Alcohol and COVID-19: What you Need to Know. https://www.euro.who.int/__data/assets/pdf_file/0010/437608/Alcohol-and-COVID-19-what-you-need-to-know.pdf?ua=1.

[31] Vetter S., Rossegger A., Rossler W., Bisson J.I., Endrass J. (2008). Exposure to the tsunami disaster, PTSD symptoms and increased substance use–An Internet based survey of male and female residents of Switzerland. BMC Public Health.

[32] Caspani M., Shumaker L. (2020). Hospitals in Wisconsin, Texas under Strain as COVID-19 Cases Surge. Reuters.

[33] Smalley C.M., Malone D.A., Meldon S.W., Borden B.L., Simon E.L., McKinsey R.M., Fertel B.S. The Impact of Covid-19 on Suicidal Ideation and Alcohol Presentations to Emergency Departments in A Large Healthcare System. https://www.ncbi.nlm.nih.gov/pmc/articles/PMC7263212/pdf/main.pdf.

[34] Campbell C.A., Hahn R.A., Elder R., Brewer R., Chattopadhyay S., Fielding J., Naimi T.S., Toomey T., Lawrence B., Middleton J.C. (2009). The effectiveness of limiting alcohol outlet density as a means of reducing excessive alcohol consumption and alcohol-related harms. Am. J. Prev. Med..

[35] Hahn R.A., Kuzara J.L., Elder R., Brewer R., Chattopadhyay S., Fielding J., Naimi T.S., Toomey T., Middleton J.C., Lawrence B. (2010). Effectiveness of policies restricting hours of alcohol sales in preventing excessive alcohol consumption and related harms. Am. J. Prev. Med..

[36] Keeter S., McGeeney K. (2015). Coverage Error in Internet surveys: Who Web-Only Surveys Miss and How That Affects Results. Pew Research Center.

[37] Dey M., Frazis H., Loewenstein M.A., Sun H. Ability to Work from Home: Evidence from Two Surveys and Implications for the Labor Market in the COVID-19 Pandemic.

[38] Knibbe R.A., Bloomfield K. (2001). Alcohol consumption estimates in surveys in Europe: Comparability and sensitivity for gender differences. Subst. Abuse.

